# Soluble Urokinase Plasminogen Activator Receptor (suPAR) Concentrations Are Elevated in Patients with Neuroendocrine Malignancies

**DOI:** 10.3390/jcm9061647

**Published:** 2020-05-31

**Authors:** Burcin Özdirik, Anna Stueven, Jana Knorr, Lukas Geisler, Raphael Mohr, Münevver Demir, Teresa Hellberg, Sven H. Loosen, Fabian Benz, Bertram Wiedenmann, Frank Tacke, Alexander Wree, Henning Jann, Christoph Roderburg

**Affiliations:** 1 Department of Hepatology and Gastroenterology, Campus Virchow Klinikum and Campus Charité Mitte, Charité University Medicine Berlin, 13353 Berlin, Germany; burcin.oezdirik@charite.de (B.Ö.); anna-kathrin.stueven@charite.de (A.S.); jana.knorr@charite.de (J.K.); lukas.geisler@charite.de (L.G.); raphael.mohr@charite.de (R.M.); muenevver.demir@charite.de (M.D.); teresa.hellberg@charite.de (T.H.); fabian.benz@charite.de (F.B.); bertram.wiedenmann@charite.de (B.W.); frank.tacke@charite.de (F.T.); alexander.wree@charite.de (A.W.); henning.jann@charite.de (H.J.); 2 Department of Medicine III, University Hospital RWTH Aachen, Pauwelsstrasse 30, 52074 Aachen, Germany; sloosen@ukaachen.de

**Keywords:** soluble urokinase-type plasminogen activator receptor, neuroendocrine tumor, neuroendocrine carcinoma, biomarker, survival

## Abstract

Neuroendocrine neoplasia (NEN) comprises heterogeneous tumors that are challenging to diagnose and, especially in cases of poorly differentiated (G3) NEN, are associated with very limited survival. Novel biomarkers allowing an early diagnosis as well as an optimal selection of suitable treatment options are urgently needed to improve the outcome of these patients. Recently, alterations of soluble urokinase-type plasminogen activator receptor (suPAR) serum levels were described in various types of cancers. However, the role of circulating suPAR as a biomarker in patients with NEN is unknown. In this study, we measured suPAR serum levels in a large and well-characterized cohort of 187 patients with NEN (neuroendocrine carcinomas (NEC) *n* = 30; neuroendocrine tumors (NET), *n* = 157) as well as 44 healthy controls. suPAR concentrations were significantly elevated in patients compared to controls. However, suPAR concentrations were independent of tumor-related factors such as the proliferation activity according to Ki-67, tumor grading, TNM (TNM classification of malignant tumors) stage, somatostatin receptor expression or clinical features such as functional or nonfunctional disease and the presence of tumor relapse. Interestingly, suPAR concentrations in NET patients were similar when compared to those measured in NEC patients. In contrast to previous results from other malignancies, in our analysis suPAR levels were not a significant predictor of overall survival. In conclusion, our data suggests that suPAR serum concentrations are elevated in NEN patients but do not allow prediction of outcome.

## 1. Introduction

Neuroendocrine neoplasms (NEN) are relatively rare and represent a very heterogeneous group of tumors. While well-differentiated neuroendocrine tumors (NET) generally have a good prognosis [1], clinical detection and diagnosis of poorly differentiated neuroendocrine carcinomas (NEC) mostly takes place at the late stages when metastatic spread has occurred and cause very poor survival times [2].

In case of a localized NEN, surgical resection represents the cornerstone of a potentially curative therapeutic approach [3,4]. If surgical tumor resection is not feasible, systemic therapy is the standard of care [3,4]. Over the last decades, new substances have emerged for systemic therapy of NEN, however, the number of available phase-III studies defining optimal treatment is limited and head-to-head studies are lacking for most situations [5,6]. While “cold” somatostatin analogues are used in almost all cases for first line treatment in well-differentiated (G1/G2), low proliferative (Ki-67 < 10%) NET, peptide receptor radionuclide therapy (PRRT) represents the gold standard for the second-line treatment (after failure of “cold” somatostatin analogues) of these patients, when tumors are positive for somatostatin receptors. Classical cytotoxic chemotherapy or molecular targeted therapy (e.g., everolimus) are used in patients with less differentiated tumors or when other treatments are not feasible (e.g., in patients without expression of the somatostatin receptor) [7,8]. As pointed out, the prognosis of patients with NEN is very heterogeneous and depending on different factors such as tumor differentiation/proliferation, tumor burden and response to therapy [9]. However, at present there are no established markers to reliably identify the ideal candidates for the different treatment modalities or to predict overall prognosis in patients with NEN [10].

The soluble urokinase plasminogen activator receptor (suPAR) is the circulating form of the cell surface receptor urokinase plasminogen activator receptor (uPAR) (CD87), which is expressed by a variety of cells including immune and epithelial cells [11,12]. suPAR was identified as a biomarker in patients with inflammatory and infectious diseases [13,14,15,16]. Moreover, a prognostic role for suPAR serum levels has been demonstrated in different cancers including pancreatic, cholangiocellular and colorectal malignancies [17,18,19,20]. However, in NET no data on a potential role of circulating suPAR as a biomarker for prediction of patients’ prognosis or response to therapies have been reported so far.

In this study, we therefore evaluated a potential role of circulating suPAR as a biomarker in a large and well characterized cohort of patients with NEN that were treated at our institution between 2010 until recently. suPAR concentrations were linked to the patients’ clinical characteristics and correlated to their outcome.

## 2. Materials and Methods

### 2.1. Design of Study and Patient Cohort

In this study, we evaluated circulating levels of suPAR as a novel diagnostic and/or prognostic biomarker in a cohort of 187 patients with NEN that were treated at our institution between 2010 until recently. suPAR concentrations were linked to the patients’ clinical characteristics and correlated to their outcome. The presence of NEN was confirmed histopathologically after biopsy or tumor resection in all cases. Patients’ blood samples were collected and were centrifuged for 10 min at 2000 *g*, and serum aliquots of 1 mL were frozen immediately at −80 °C in order to avoid repetitive freeze-thaw cycles until use. 44 healthy, cancer-free blood donors served as control samples. The study protocol was approved by the ethics committee of Charité, University Medicine Berlin, Germany (ethical approval number EA1/229/17). Written informed consent was obtained from all patients.

### 2.2. Measurement of Circulating suPAR Levels

Serum levels of suPAR were measured with an enzyme-linked immunosorbent assay (ELISA) according to the manufacturers’ instructions (Nr. A001, suPARnostic, ViroGates, Birkerød, Denmark). In detail, 25 µL of each suPAR standard and 25 µL of each serum specimen were mixed with 225 µL Peroxidase Conjugate solution in a mixing plate. In a next step, 100 µL of the mixed samples were transferred in duplicates into wells of a new clear-coated plate. The plate was covered with sealing tape to prevent evaporation and was incubated at room temperature (18–26 °C) in the dark. After an incubation period of one hour, the wells were washed five times with Wash Buffer (250 µL per well). In a next step, 100-µL 5′-tetramethylbenzidine (TMB) solution was added to each well and incubated for twenty minutes at room temperature. The reaction was stopped by adding 100 µL of stop solution. Evaluation of the ELISA absorbance values and calculation of the serum concentration were performed using a 4-parameter logistic nonlinear regression model. Standard laboratory parameters were measured at Labor Berlin, the central laboratory of Charité, University Medicine Berlin, Germany. Serum samples were measured without previous dilution.

### 2.3. Statistical Analysis

Serum data are displayed as Boxplots. Nonparametric data were compared using the Mann–Whitney *U*-test or the Kruskal–Wallis test for multiple group comparisons. Correlation analyses were performed using the Spearman’s correlation coefficient. Column bar graphics display the ranges. We generated receiver operating characteristic (ROC) curves by plotting the sensitivity (%) against 100%-specificity (%). Kaplan–Meier curves display the impact of a specific parameter on the overall survival. The respective 95% confidence intervals (CI) were estimated with the Kaplan–Meier survival method. Survival curves between groups were compared by the log–rank Mantel–Cox test. All statistical analyses were performed with Prism (version 7.03; GraphPad, La Jolla, California, USA). A *p*-value of <0.05 was considered statistically significant.

## 3. Results

### 3.1. Patients’ Characteristics of the Two NEN Cohorts

One hundred fifty-seven patients with histologically confirmed NET were included into the present analysis. Out of these, 82 (52%) were male and 75 (48%) female with a median age of 46 years (30–80) at initial diagnosis. Primary tumor localization was as following: ileum (*n* = 81), pancreas (*n* = 73), lung (*n* = 1), ovary (*n* = 1). The median time of follow-up was 16 years (range 3–28 years) and the median Ki-67 proliferation index was 2 (range 1–25). Moreover, we analysed a cohort of patients with NEC (*n* = 30). Out of these, 18 (60%) were male and 12 (40%) were female with a median age of 49 years (range 26–71) at initial diagnosis. Again, primary tumor localization was mainly of gastroenteropancreatic origin: pancreas (*n* = 14), stomach (*n* = 7), ileum (*n* = 2), rectum (*n* = 2), thymus (*n* = 1), lung (*n* = 1), larynx (*n* = 1), cortex of the suprarenal gland (*n* = 1) and one NEC of unknown origin. The median time of follow-up was 10 years (range 0–15 years). Median Ki-67 proliferation index was 23 (20–95). Patients’ characteristics are summarized in Table 1 and Table 2.

### 3.2. Circulating Levels of suPAR Are Elevated in NET Patients

**Figure 1:**
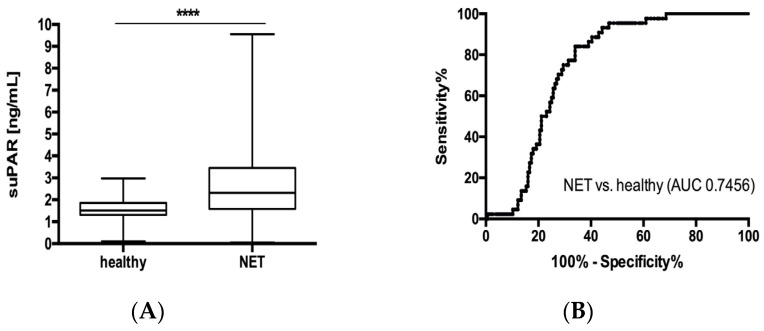

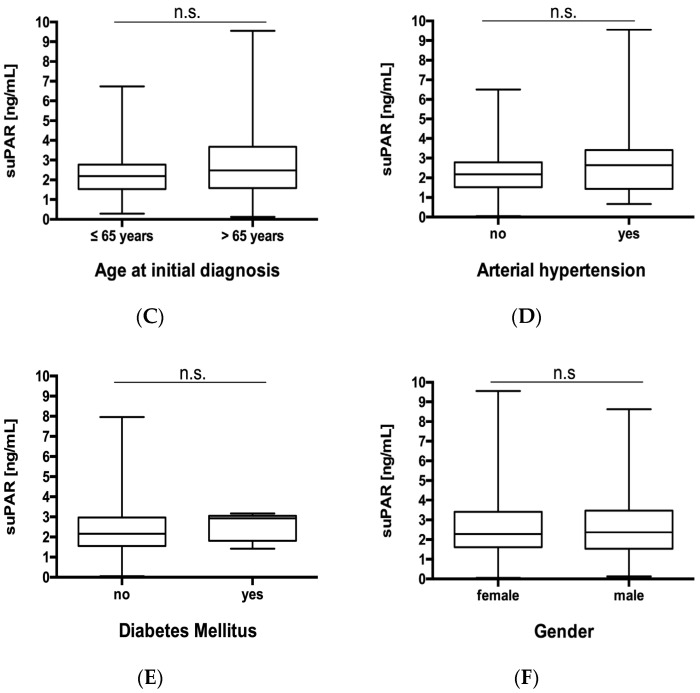
Serum suPAR levels are elevated in NET patients. (**A**) Serum concentrations of suPAR are significantly elevated in patients with NET compared to healthy controls. (**B**) suPAR levels display an AUC value of 0.7456 regarding the discrimination of NET patients and healthy controls. SuPAR levels were similar in patients (**C**) with or without arterial hypertension (**D**) with or without type 2 diabetes and (**E**) patients younger/ older than 65 years and (**F**) did not vary with respect to patients’ gender. Box plot are displayed, where the bold line indicates the median per group, the box represents 50% of the values. The horizontal lines show minimum and maximum values of calculated nonoutlier values (**** *p* < 0.0001). NET, neuroendocrine tumors; suPAR, soluble urokinase-type plasminogen activator receptor; AUC, area under the curve.

Elevated levels of suPAR were recently described in very different pathologies, including gastrointestinal malignancies [21,22,23]. To analyse whether suPAR serum levels are also altered in patients with NET, we first compared levels of circulating suPAR between samples from 157 patients with neuroendocrine tumors and samples from 44 healthy controls. This analysis revealed significantly elevated suPAR serum concentrations in the NET patient group compared to healthy controls (Figure 1A). To analyse the power of suPAR to distinguish between patients with NET and those without, we next performed ROC curve analyses. These analyses showed an area under the curve (AUC) of 0.7456 for suPAR regarding the discrimination between NET and healthy controls (Figure 1B). At the ideal cut-off value of 1.995 pg/mL, suPAR showed a sensitivity of 84% and a specificity of 66% for identification of NET.

Alterations in suPAR concentrations were recently demonstrated in the context of metabolic and cardiovascular diseases [24,25]. We therefore analyzed whether type 2 diabetes mellitus or arterial hypertension might have an influence on circulating suPAR in patients with NET. However, in our cohort suPAR levels were independent of the presence of these metabolic comorbidities (Figure 1C,D). Finally, levels of circulating suPAR were independent of the patient’s age or sex (Figure 1E,F).

### 3.3. suPAR Serum Concentrations Are not Associated with Disease Characteristics in Patients with NET

**Figure 2:**
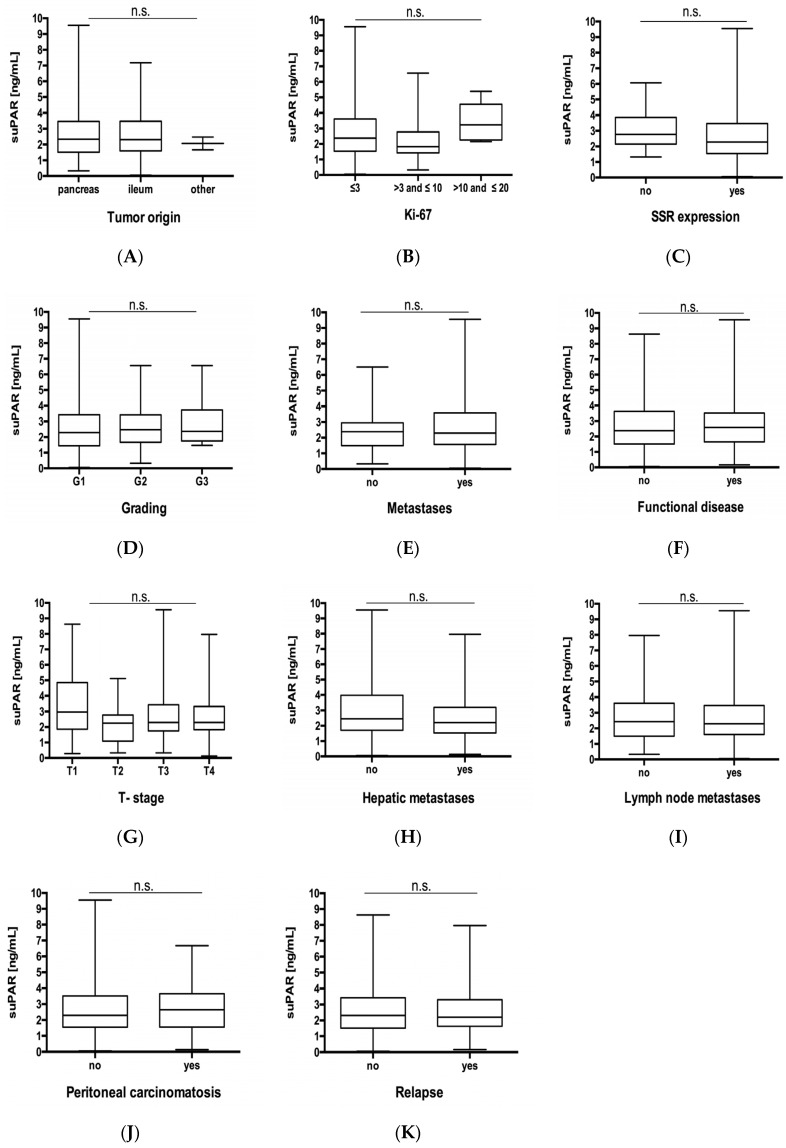
suPAR serum levels do not reflect tumor characteristics. Circulating levels of suPAR are unaltered between NET patients with (**A**) different tumor localization, (**B**) with lower or higher Ki-67 rates, (**C**) histological grade (Grade (G) 1 to 3), (**D**) functional or nonfunctional disease, (**E**) SSR positive or negative disease as well SSR positive or negative disease. Furthermore, analysis of different subgroups with. (**F**) different T-stages, (**G**) non-metastasized and metastasized patients, (**H**) lymph node metastases as well as (**I**) hepatic and (**J**) peritoneal carcinomatosis did not show any significant difference. (**K**) postoperative relapse status did not show any significant difference. Box plot are displayed, where the bold line indicates the median per group, the box represents 50% of the values. The horizontal lines show minimum and maximum values of calculated nonoutlier values. NET, neuroendocrine tumors; SSR, Somatostatin receptor.

Based on these results, suggesting a role for suPAR as a biomarker in NET, we analyzed whether serum levels of suPAR might reflect disease specific clinicopathological characteristics such as tumor localization, tumor proliferation rate, tumor burden, histological tumor grading, functional/nonfunctional disease and somatostatin receptor (SSTR)-expression. We therefore performed subgroup analyses and specifically analyzed suPAR concentrations in patients with different tumor localization (Figure 2A) with lower or higher Ki-67 rates (Figure 2B), histological grade 1 to 3 tumors (Figure 2C), functional or non-functional disease (Figure 2D) as well as positive or negative somataostatin receptor (SSR) expression status (Figure 2E). Furthermore, we compared more advanced or earlier disease (Figure 2F), nonmetastasized or metastasized disease (Figure 2G), lymph node positive or negative disease stage (Figure 2H), as well as subgroups with/ without hepatic- (Figure 2I) and peritoneal carcinomatosis (Figure 2J). However, no differences in suPAR concentrations between all these different subgroups were apparent. Finally, we analyzed whether suPAR concentrations might reflect postoperative relapse status (Figure 2K). However, also in these analyses no significant differences in suPAR serum levels were found.

**Figure 3:**
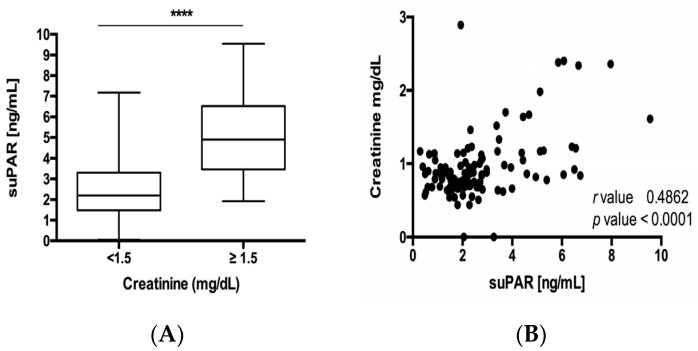
suPAR levels in NET are indicative for renal failure. (**A**) Serum levels of suPAR were significantly higher in patients with elevated serum creatinine. (**B**) Both parameters demonstrated a significant correlation according to spearman rank correlation analysis (Figure 3B). Box plot are displayed, where the bold line indicates the median per group, the box represents 50% of the values. The horizontal lines show minimum and maximum values of calculated nonoutlier values (**** *p* < 0.0001).

Circulating suPAR has recently been associated with chronic kidney disease and elevated suPAR serum levels were suggested to predict impaired renal function after cardiac surgery [26,27,28]. Hence, we evaluated if circulating levels of suPAR might be indicative for kidney injury in our cohort of patients. Interestingly, serum levels of suPAR were significantly higher in patients with elevated serum creatinine concentrations (Figure 3A) and both parameters demonstrated a significant correlation according to Spearman rank correlation analysis (Figure 3B).

### 3.4. suPAR Serum Concentrations Are not Associated with Overall Survival in Patients with NET

**Figure 4:**
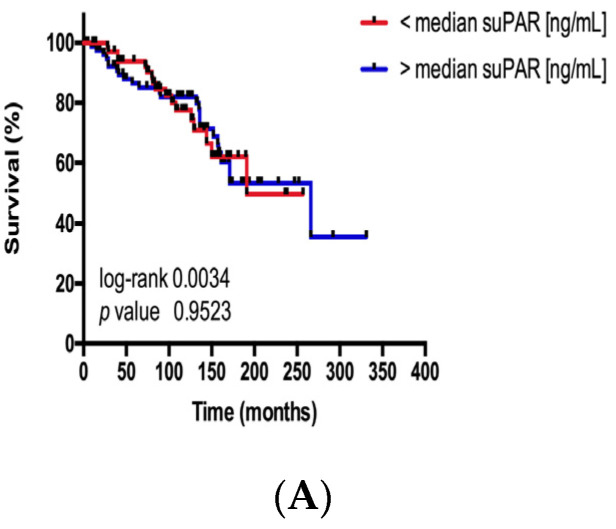

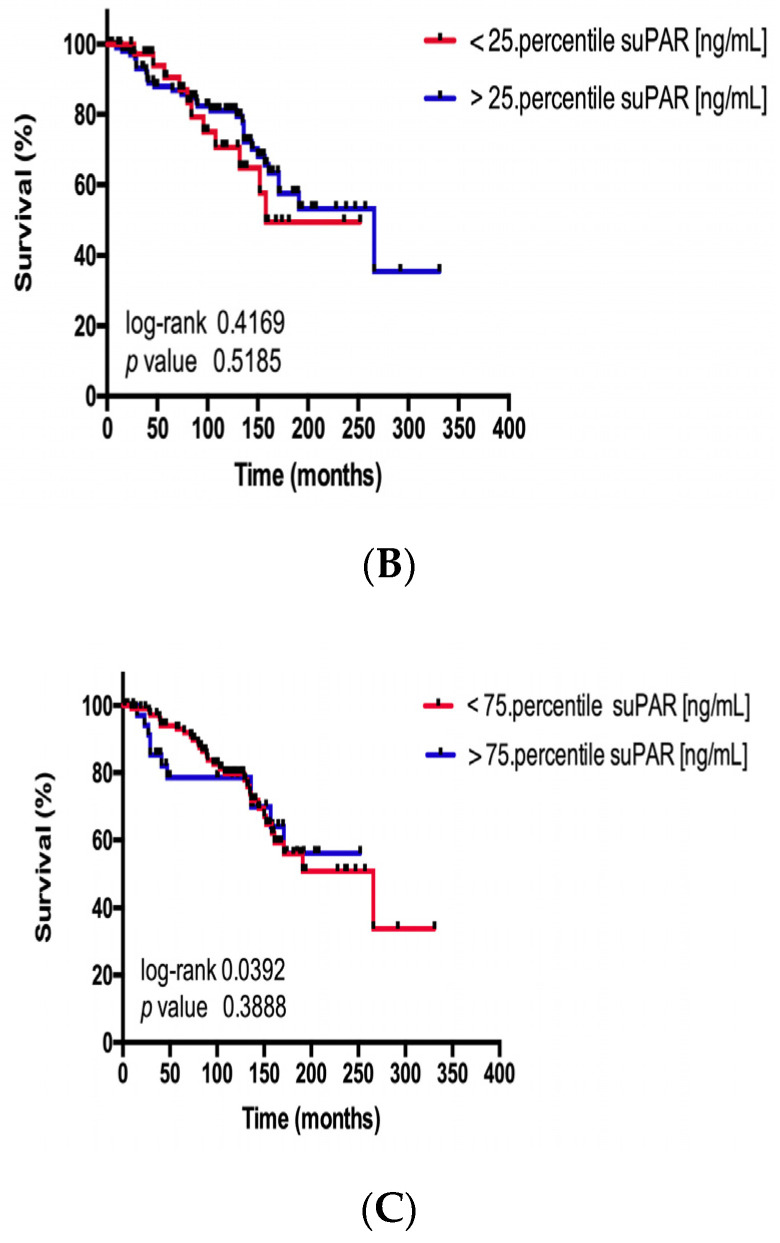
suPAR serum levels are not associated with the patients’ prognosis. Kaplan–Meier analysis of serum suPAR levels above (red curve) and below (blue curve) the (**A**) median (2.31 ng/mL), (**B**) the 25th percentile (1.58 ng/mL) and (**C**) 75th percentile (3.46 ng/mL) show a similar overall survival. Consequently, the median, 25th and 75th percentile are not appropriate cut-off values of suPAR serum levels to discriminate between patients with a good or poor long-term prognosis.

A prognostic role of suPAR serum concentrations was recently suggested in patients with benign and malignant diseases. Based on these data, we aimed to validating the prognostic relevance of circulating suPAR in our cohort of patients with NET. In this analysis, the subgroup of patients with suPAR serum concentrations above/below the median, 25th or 75th percentile of all patients demonstrated a similar survival. Thus, at least in our cohort of patients, suPAR did not represent a prognostic marker (Figure 4A–C).

### 3.5. suPAR Serum Concentrations are Similarly Elevated in Patients with Neuroendocrine Tumors and Neuroendocrine Carcinoma

**Figure 5:**
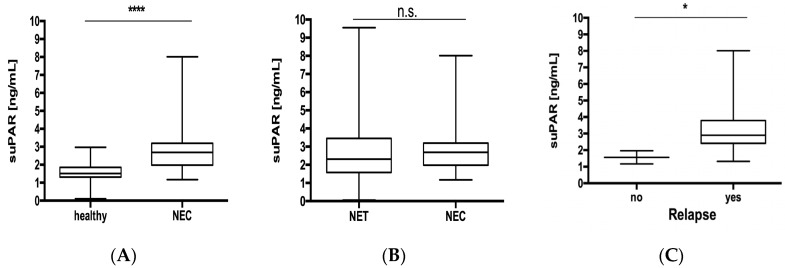
suPAR levels do not discriminate between neuroendocrine tumors and neuroendocrine carcinoma. (**A**) Serum concentrations of suPAR are significantly elevated in patients with NEC compared to healthy controls. (**B**) suPAR levels were similar in patients with NEC and NET. (**C**) NEC patients with disease relapse after surgery displayed elevated suPAR concentrations. Box plot are displayed, where the bold line indicates the median per group, the box represents 50% of the values. The horizontal lines show minimum and maximum values of calculated nonoutlier values (* *p* < 0.05, **** *p* < 0.0001). NEC, neuroendocrine carcinomas.

As pointed out before, neuroendocrine tumors and neuroendocrine carcinoma represent different diseases in terms of their tumor biology, therapy and prognosis. Therefore, we additionally analyzed suPAR concentrations in 30 patients that fulfilled the WHO criteria for neuroendocrine carcinoma. While patients with NEC displayed elevated suPAR concentrations when compared to healthy controls (Figure 5A), suPAR concentrations were similar in patients with NEC and NET (Figure 5B). Similar to our analyses from NET, suPAR levels did not reflect patients’ characteristics or clinicopathological features (Figure S1). Interestingly, NEC patients did not show a significant correlation when comparing creatinine and suPAR levels (Figure S1) as we have found out in NET patients. Presumably, this can be led back to the lower number of NEC patients included in this study. Despite NEC patients without tumor relapse after surgery displayed lower levels of circulating suPAR (Figure 5C), concentrations of suPAR were not associated to patient’s survival since patients with suPAR serum concentrations above/below the median, 25th or 75th demonstrated a similar survival (Figure S2).

## 4. Discussion

We demonstrate that serum concentrations of suPAR are elevated in patients with neuroendocrine neoplasia but neither reflect specific clinicopathological characteristics nor patient’s survival.

Neuroendocrine neoplasia represents a rare but sometimes highly aggressive type of cancer [29,30]. Surgical tumor resection is the only available curative treatment option but the patients’ long-term outcome, even after successful tumor resection, is often limited due to high risk of recurrence [31,32]. At present, the only clinically available tumor marker, Chromogranin A (CgA), has been demonstrated to be of limited sensitivity and specificity and is in most cases only used to monitor tumor response to chemotherapy [10]. By analyzing a large and well characterized cohort of 157 patients with neuroendocrine tumors, we demonstrated that serum concentrations of suPAR were elevated in patients with neuroendocrine neoplasia. Of note, suPAR concentrations were independent on patients’ age or sex, highlighting the stability of this marker in the context of NET. Given the low incidence of NET, analysis from large and clinically well annotated patients’ cohorts are scarce. In this context, the data presented here are potentially of high interest, since diagnosis of NEN still relies on histopathological analysis. This stands in contrast to other malignancies, where serum based markers (“liquid biopsy“) were proposed as an easy alternative to histology, allowing tumor diagnosis and estimation of patients’ prognosis without invasive biopsy [33]. Our data suggest that suPAR could represent a promising candidate when evaluating blood-based biomarkers for diagnosing NET. Testing a combination of CgA and suPAR for diagnosis of NET might represent an interesting task in the context of neuroendocrine neoplasia.

Our data are in line with previous publications showing elevated suPAR serum levels in manifold cancers. Just recently, we demonstrated that concentrations of suPAR are elevated in patients with cholangiocellular carcinoma (CCA) [18] as well as in patients with pancreatic ductal adenocarcinoma (PDAC) [34] or colorectal cancer (CRC) [20]. Notably, serum concentrations of suPAR in our cohort of patients with NET are numerically very similar to those found in CCA, PDAC and CRC. Interestingly, in all of these entities, despite being linked to patients’ survival, suPAR serum levels were not associated with a more advanced disease stage, corroborating the data shown within this manuscript. However, in contrast to the above mentioned types of adenocarcinomas, suPAR concentrations in patients with NET did not reflect patients’ survival. Moreover, suPAR concentrations were not linked to the tumor localization nor to the tumor proliferation activity, highlighting that in patients with NET suPAR serum levels rather reflect a general systemic response to the presence of a malignant tumor than specific clinicopathological features. This clearly raises the question on the source of elevated suPAR concentrations in the context of NET. It is presently unknown whether primary NET and/or its metastases show a strong uPAR expression. Our own preliminary data suggest that not the tumor itself but rather infiltrating immune cells demonstrate positivity for uPAR by immunohistochemical analysis. This findings would be in line to data from CRC showing that intratumoral uPAR predominately origins from infiltrating macrophages and neutrophils and only to a much lesser extend from malignant tumor cells [35]. The data fit to observations from patients with nonmalignant inflammatory diseases, in which neutrophils and monocytes are the most important origin of suPAR [36,37]. Given the missing association of suPAR serum levels with tumor grading or staging, it is likely that elevated suPAR serum levels in NET patients originate from an increased shedding of uPAR in immune cells and not from tumor cells themselves.

Neuroendocrine neoplasia comprise both neuroendocrine tumors and neuroendocrine carcinoma, which however, are fully different tumor entities [3,34,35]. In addition to NET, we therefore analyzed suPAR levels within a large cohort of patients with NEC. Interestingly, suPAR concentrations were elevated when compared to healthy controls but similar to those found in patients with NET. This corroborates the notion that suPAR rather represents a general disease marker than an entity specific biomarker in the context of gastrointestinal malignancies.

## 5. Conclusions

In summary, our data suggest that suPAR measurements in serum might be useful as an additional tool in the complex diagnostic work-up of patients with NET. However, it is important to note that our study bears some important limitations. Although the single center design of our study implicates a comparability of included patients with respect to eligibility criteria and applied treatment algorithms, this design warrants a confirmation in a multi-center approach. Moreover, our study did not include longitudinal measurements during treatment such as chemotherapy or loco-regional therapies. Thus, we cannot answer the decisive question, whether the course of suPAR concentrations might be predictive for tumor response and whether patients with a decrease in suPAR levels under therapy might have a better outcome than others. Therefore, further multi-center clinical trials, including larger patient numbers, are needed to gain full insight into the pathophysiological and clinical importance of suPAR in the context of NET.

## Figures and Tables

**Table 1:** NET patients’ characteristics.

Characteristics	All Patients
	*n* = 157 (100%)
Sex, female	75 (48%)
Age at initial diagnosis, median	46 (36–80)
**Comorbidities**	***n* = 86**
Diabetes	*n* = 15 (17%)
Arterial hypertension	*n* = 20 (23%)
**Primary tumor localization**	
ileum	*n* = 82 (52%)
pancreas	*n* = 73 (46%)
Other:	*n* = 2 (1%)
Lung	*n* = 1
Ovary	*n* = 1
**Median survival (months), range**	***n* = 188 (42–331)**
No. of patients alive	*n* = 54 (34%)
No. of death patients	*n* = 43 (27%)
No. of patients lost-to-follow-up	*n* = 60 (38%)
**Grading**	
G1	*n* = 83 (55%)
G2	*n* = 57 (36%)
G3	*n* = 12 (0.6%)
**Ki-67 (%), median (range)**	**2 (1–25)**
Ki-67 ≤ 3	*n* = 88 (64%)
Ki-67 > 3 and ≤ 10	*n* = 41 (30%)
Ki-67 > 10 and ≤ 20	*n* = 9 (7%)
**Metastases**	***n* = 122 (80%)**
Hepatic metastases	*n* = 20 (83%)
Lymph node metastases	*n* = 16 (64%)
Bone metastases	*n* = 6 (27%)
Peritoneal carcinomatosis	*n* = 7 (32%)
**T-stage**	
T1	*n* = 9 (8%)
T2	*n* = 31 (26%)
T3	*n* = 45 (38%)
T4	*n* = 35 (29%)
**Relapse**	
no	*n*= 53 (71%)
yes	*n* = 22 (29%)
**Functional disease**	
no	*n* = 77 (53%)
yes	*n* = 68 (47%)
**SSR expression**	
no	*n* = 22 (16%)
yes	*n* = 117 (84%)
**Creatinine (mg/dL) median (range)**	**0.85 (0.44–2.89)**
<1.5 mg/dL	*n* = 126 (92%)
≥1.5 mg/dL	*n* = 10 (7%)
**Chromogranin A, median (range)**	**97 (0–7986)**
<97 μg/L	*n* = 46 (49%)
≥97 μg/L	*n* = 48 (51%)

NET, neuroendocrine tumors; SSR, somatostatin receptor.

**Table 2:** NEC patients’ characteristics.

Characteristics	All Patients (*n* = 30)
Sex, female	12 (40%)
Age at initial diagnosis, median (years)	49 (26–71)
**Comorbidities**	
Diabetes	*n* = 3 (27%)
Arterial hypertension	*n* = 3 (27%)
**Primary tumor localization**	
Pancreas	*n* = 14 (47%)
Ileum	*n* = 2 (6%)
Stomach	*n* = 7 (23%)
Other:	*n* = 7 (23%)
Rectum	*n*= 2
Thymus	*n* = 1
Lung	*n* = 1
Larynx	*n* = 1
Cortex of suprarenal gland	*n* = 1
CUP	*n* = 1
**Median survival (months), range**	***n* = 123 (0–176)**
No. of patients alive	*n* = 11 (37%)
No. of death patients	*n* = 7 (23%)
No. of patients lost-to-follow-up	*n* = 12 (40%)
**Metastases**	***n* = 20**
Hepatic metastases	*n* = 20 (20/24; 83%)
Lymph node metastases	*n* = 16 (16/25; 64%)
Bone metastases	*n* = 6 (6/22; 27%)
Peritoneal carcinomatosis	*n* = 7 (7/22; 32%)
**Grading:**	***n* = 29**
G1	*n* = 0
G2	*n* = 2 (7%)
G3	*n* = 25 (93%)
**Ki-67 (%), median (range)**	**28 (20–95)**
Ki-67 ≤ 20	*n* = 8 (36%)
Ki-67 >20	*n* = 14 (64%)
**T stage**	***n* = 9**
T1	*n* = 0
T2	*n* = 2 (22%)
T3	*n*= 3 (33%)
T4	*n* = 4 (44%)
**Relapse**	
no	*n* = 19 (95%)
yes	*n* = 1 (5%)
**Functional disease**	
no	*n* = 16 (80%)
yes	*n* = 4 (20%)
**SSR expression**	***n* = 22**
no	*n* = 16 (73%)
yes	*n* = 6 (27%)
**Creatinine (mg/dL), median; (range)**	**0.85; (0.52–1.60)**
Creatine < 1	*n* = 21 (81%)
Creatine ≥ 1	*n* = 5 (19%)
**Chromogranin A (μg/L), median; (range)**	**138 (1–454)**
<137 μg/L	*n* = 4 (50%)
≥137 μg/L	*n* = 4 (50%)

NEC, neuroendocrine carcinomas; CUP, cancer of unknown primary; SSR, somatostatin receptor.

## Supplementary Materials

The following are available online at https://www.mdpi.com/2077-0383/9/6/1647/s1, Figure S1: suPAR serum concentrations are similar in patients with neuroendocrine tumors and neuroendocrine carcinoma. Circulating levels of suPAR in NEC did not reflect (A) gender, (B) age, metabolic comorbidities such as (C) diabetes and (D) arterial hypertension. Circulating levels of suPAR did not reflect clinicopathological tumor characteristics such as (E) tumor origin, (F) proliferation rate Ki-67 and (G) presence of metastases. (H) Moreover, we did not find any significant correlation between suPAR levels and creatinine values. Box plot are displayed, where the bold line indicates the median per group, the box represents 50% of the values. The horizontal lines show minimum and maximum values of calculated non-outlier values; Figure S2: suPAR serum levels in patients with neuroendocrine carcinoma are not associated with the patients’ prognosis. Kaplan-Meier analysis of serum suPAR levels above (red curve) and below (blue curve) the (A) median (2.69 ng/mL), (B) the 25th percentile (1.98 ng/mL) and (C) 75th percentile (3.2 ng/mL) show a similar overall survival. Consequently, the median, 25th and 75th percentile are not appropriate cut-off values of suPAR serum levels to discriminate between NEC patients with a good or poor long-term prognosis.

## Abbreviations

5′-tetramethylbenzidine	TMB
Area under the curve	AUC
Cholangiocellular carcinoma	CCA
Colorectal Cancer	CRC
Confidence Interval	CI
Chromogranin A	CgA
Enzyme-linked immunosorbent assay	ELISA
Grade	G
Neuroendocrine carcinoma	NEC
Neuroendocrine neoplasm	NEN
Neuroendocrine tumors	NET
Odds Ratio	OR
Overall survival	OS
Pancreatic ductal adenocarcinoma	PDAC
Peptide receptor radionuclide therapy	PRRT
Receiver operating characteristic	ROC
Soluble urokinase-type plasminogen activator receptor	suPAR
Somatostatin receptor	SSR
Temozolomide/Capecitabine	TEM/CAP
TNM classification of malignant tumors	TNM
Urokinase plasminogen activator receptor	uPAR
