# Supplementary material for: Soluble Urokinase Plasminogen Activator Receptor (suPAR) Concentrations Are Elevated in Patients with Neuroendocrine Malignancies
Source: J Clin Med. 2020 May 31;9(6):1647. doi: 10.3390/jcm9061647 (PMC7355627; doi:10.3390/jcm9061647)
Supplement: Supplementary file 1 [file jcm-09-01647-s001.pdf]

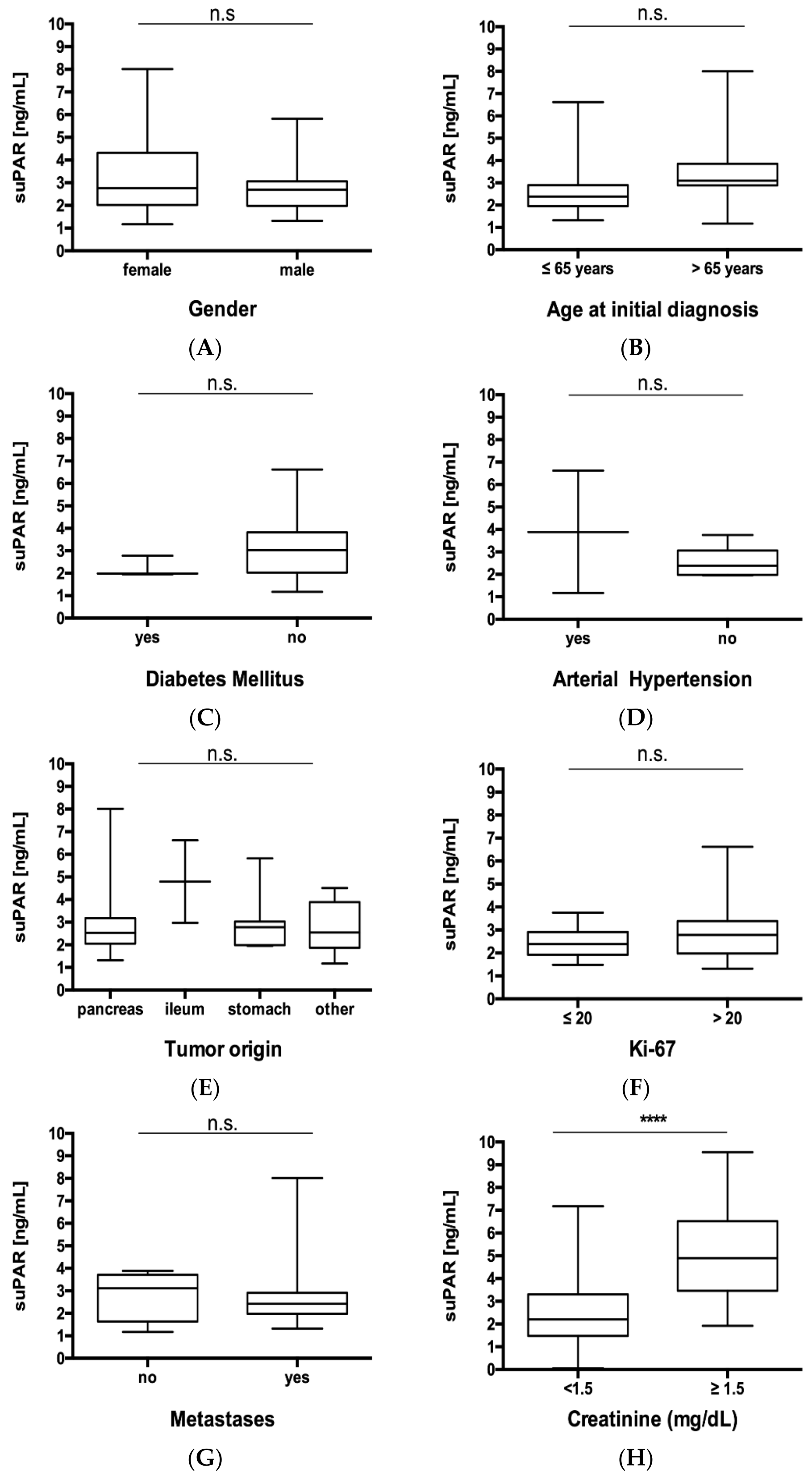

Figure S1. suPAR serum concentrations are similar in patients with neuroendocrine tumors and neuroendocrine carcinoma. Circulating levels of suPAR in NEC did not reflect (**A**) gender, (**B**) age, metabolic comorbidities such as (**C**) diabetes and (**D**) arterial hypertension. Circulating levels of suPAR did not reflect clinicopathological tumor characteristics such as (**E**) tumor origin, (**F**) proliferation rate Ki-67 and (**G**) presence of metastases. (**H**) Moreover, we did not find any significant correlation between

suPAR levels and creatinine values. Box plot are displayed, where the bold line indicates the median per group, the box represents 50% of the values. The horizontal lines show minimum and maximum values of calculated non-outlier values. suPAR, soluble urokinase-type plasminogen activator receptor; NEC, neuroendocrine carcinomas.

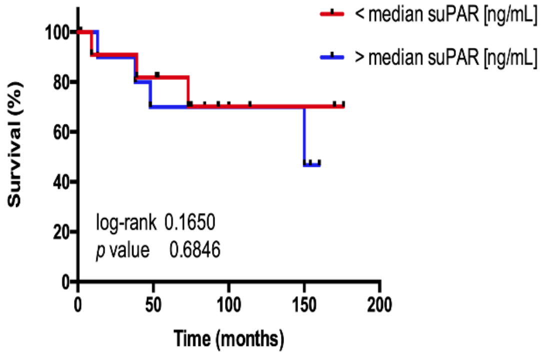

(A)

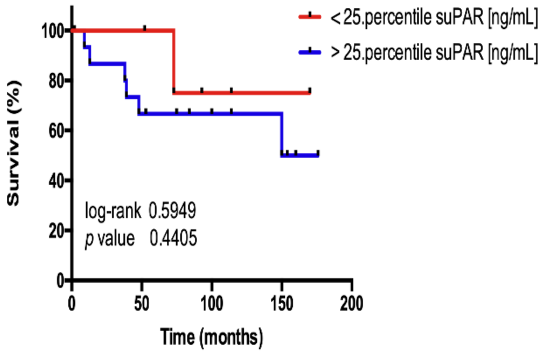

(B)

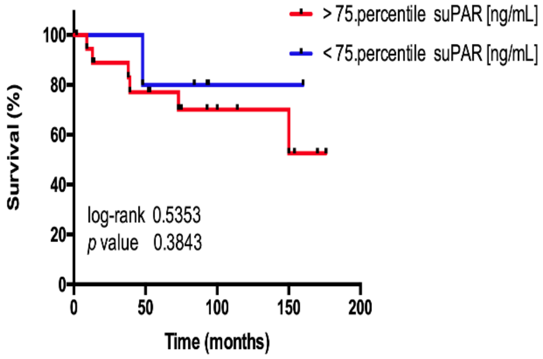

(C)

Figure S2. suPAR serum levels in patients with neuroendocrine carcinoma are not associated with the patients' prognosis. Kaplan-Meier analysis of serum suPAR levels above (red curve) and below (blue curve) the (**A**) median (2.69 ng/mL), (**B**) the 25th percentile (1.98 ng/mL) and (**C**) 75th percentile (3.2 ng/mL) show a similar overall survival. Consequently, the median, 25th and 75th percentile are not appropriate cut-off values of suPAR serum levels to discriminate between NEC patients with a good or poor long-term prognosis.
